# Performance on cognitive tests, instrumental activities of daily living and depressive symptoms of a community-based sample of elderly adults in Rio de Janeiro, Brazil

**DOI:** 10.1590/1980-57642016dn11-010009

**Published:** 2017

**Authors:** Christina Martins Borges Lima, Heloisa Veiga Dias Alves, Daniel Correa Mograbi, Flávia Furtado Pereira, Jesus Landeira Fernandez, Helenice Charchat-Fichman

**Affiliations:** 1 Pontificia Universidade Catolica do Rio de Janeiro - Psicologia, Rio de Janeiro RJ – Brazil.; 2 Bolsista do CNPq.; 3 Secretaria Especial de Envelhecimento e Qualidade de Vida – SESQV, Rio de Janeiro RJ – Brazil.

**Keywords:** aging, elderly, cognition, neuropsychological tests, Brief Cognitive Screening Battery

## Abstract

**Objective:**

To describe the performance on basic cognitive tasks, instrumental activities of daily living, and depressive symptoms of a community-based sample of elderly adults in Rio de Janeiro (Brazil) who participated in multiple physical, social, and cognitive activities at government-run community centers.

**Methods:**

A total of 264 educated older adults (> 60 years of age of both genders) were evaluated by the Brief Cognitive Screening Battery (BCSB), Lawton's and Pfeffer's activities of daily living indexes, and the Geriatric Depressive Scale (GDS).

**Results:**

The mean age of the sample was 75.7 years. The participants had a mean of 9.3 years of formal education. With the exception of the Clock Drawing Test (CDT), mean scores on the cognitive tests were consistent with the values in the literature. Only 6.4% of the sample had some kind of dependence for activities of daily living. The results of the Geriatric Depression Scale (GDS-15) indicated mild symptoms of depression in 16.8% of the sample

**Conclusion:**

This study provided important demographic, cognitive, and functional characteristics of a specific community-based sample of elderly adults in Rio de Janeiro, Brazil.

## Abstract (pt)

**Objetivo:**

Descrever o desempenho em testes cognitivos, atividades instrumentais da vida diária e sintomas depressivos em uma comunidade de idosos participantes de atividades físicas, sociais e cognitivas em centros de convivência da cidade do Rio de Janeiro, Brasil.

**Métodos:**

Foram avaliados 264 idosos acima de 60 anos, de ambos os sexos e escolarizados, através de uma Bateria Breve de Rastreio Cognitiva (BBRC), escalas funcionais de atividades instrumentais Lawton e Pfeffer, bem como Escala de Depressão Geriátrica (GDS).

**Resultados:**

Os dados descritivos indicaram que a idade média da amostra foi 75,7 anos e a escolaridade média 9,3 anos. Com exceção do Teste do Desenho do Relógio (TDR), as médias dos testes cognitivos ficaram dentro dos valores encontrados com grupos similares. No desempenho funcional detectou-se independência e autonomia nas atividades cotidianas. Os resultados da Escala de Depressão Geriátrica (EDG-15) evidenciaram sintomas leves de depressão em 16,8% da amostra.

**Conclusão:**

Este estudo mostrou as características demográficas, cognitivas, funcionais de uma amostra de idosos residentes da cidade do Rio de Janeiro, Brasil que realizam múltiplas atividades em centros comunitários.

## INTRODUCTION

Population aging is a worldwide phenomenon, characterized by a higher rate of growth among the elderly population (aged ≥60 years) relative to other age groups.^1^ Currently, Brazil has more than 19.6 million people over the age of 60, representing 12% of the population, but projections indicate that this number is set to exceed 41.5 million by 2030.2

Population aging poses new challenges for society and governments in developing countries, especially with regard to public policies, which need to be adapted to this new social reality. The phenomenon of population aging needs to be described and studied in greater depth to improve current approaches for preventing and detecting the most prevalent diseases associated with the aging process.^1,2^

The first studies on aging and cognition sought to describe the decline of cognitive abilities in senescence as a natural process that involves neuropsychological changes.^3^ It is widely acknowledged that cognitive decline impacts various domains, such as memory, attention, language, and executive function, and may also influence activities of daily living and be associated with mood disorders (e.g., anxiety) and dementia.^4^

Population studies have sought to identify factors that influence normality and pathology in aging to better understand the cognitive and functional profiles of elderly adults. Ribeiro et al.^5^ found that education, age, gender, and marital status were factors that led to significant differences in cognitive performance in community-dwelling older adults. Furthermore, in a 3-year community study, Pereira et al.^6^ highlighted the importance of a cognitive reserve in aging and listed the following factors as playing a positive role in maintaining healthy cognition and independence: education, engagement in community activities, and a healthy lifestyle.

In addition to sociodemographic factors, cognitive impairment might be secondary to depressive episodes. Previous studies have reported a high incidence of depression during the aging process.^7,8^ According to the literature, depressive symptoms affect cognitive skills and can sometimes cause mild cognitive impairment.^7^ Depressive symptoms also affect functioning, making it crucial to identify and treat this pathology in this age group.

Aging itself leads to a gradual, progressive decline in functional capacity. Elderly people with normal cognitive functioning have, as a main characteristic, the maintenance of autonomy and independence. These two variables can change during the aging process and are very often related to cognitive decline and depressive symptoms, especially in populations with lower levels of education.^9^ Cognitive decline affects functional capacity and the proper performance of everyday activities.^5^ Studies have found that memory and executive dysfunction predict functional disabilities through a decline in performance of instrumental activities of daily living (IADL). Functioning in IADL is an objective measure that helps identify those individuals with increased risk of MCI and dementia.^4,7,10^

For a comprehensive understanding of healthy aging, it is essential to investigate the cognition-depression-functioning triad in community-dwelling elderly adults. Recent studies involving older adults from the community and using a screening battery to assess normal aging in this heterogeneous population, found that sociodemographic variables, particularly age, low levels of education, and symptoms of depression can strongly contribute to cognitive decline and impact functioning in these individuals.^6,8,10-12^ However, studies evaluating the diversity of the Brazilian community-based population using similar measures failed to investigate the performance of normal, healthy participants.^13,14^ In other words, previous studies only described the demographic profile and prevalence of dementia and did not report normative data on cognition and functioning. In this context, the objective of the present study was to describe the performance of a community-based sample of elderly adults on basic cognitive tasks, instrumental activities of daily living, and depressive symptoms. We sought to describe the cognitive-depressive-functional profiles of independent elderly individuals who participated in multiple physical, social, and cognitive activities offered by the government of the city of Rio de Janeiro, Brazil.

## METHODS

**Sample.** The present study evaluated 264 community-dwelling elderly adults who participated in activities offered by the government in at least one of six locations: Penha, Botafogo, Tijuca, Gávea, Sao Conrado (Rocinha), and Lagoa. The study included educated elderly individuals of both genders over 60 years of age that went every day or twice a week to do physical or cognitive training. To be included in the study, participants had to be literate and independent (i.e., not accompanied by a caregiver). In cases of chronic disease, such as hypertension, diabetes mellitus, thyroid disease, and heart disease, the participants were included only if the disease was under medical control. The exclusion criteria were:

1. presence of severe sensory deficits without correction;
2. motor or cognitive abnormalities that prevented full implementation of the assessment protocol;
3. neurological diseases such as: dementia, Parkinson's disease, and stroke;
4. use of psychotropic drugs; and
5. dependence on caregiver assistance.

**Ethical aspects.** This study was approved by the Ethics Committee of the Federal University of the State of Rio de Janeiro (UNIRIO; 1023155). All of the participants signed an informed consent form according to Resolution 196/96 of the National Health Council, which deals with the guidelines and standards for research involving humans.

**Procedures.** A standardized questionnaire was applied to characterize sociodemographic and clinical aspects. The following variables were collected: age, gender, education, marital status, ethnicity, profession, general health index, subjective cognitive decline, self-reported diseases, and the use of medications, All of the data were collected during individual interviews in a single session that lasted approximately 1 hour. All of the participants underwent the same functional evaluation protocol with validated cognitive and functional assessment instruments. The protocol was applied by qualified neuropsychologists and undergraduate psychology research assistants who were trained on the application and scoring of the instruments.

**Instruments.** The Brief Cognitive Screening Battery (BCSB), based on Nitrini et al.^15^ and subsequently validated for Brazilian samples in other studies, was employed.^4,8,16,18^ The BCSB has been shown to accurately diagnose cognitive impairment in populations with a heterogeneous educational background.^16,18^ The BCSB comprises seven instruments. The tests were administered using standardized stimuli. Responses and observations were recorded with pencil and paper. The execution time was recorded, when appropriate, with a stopwatch.

Instruments were applied in the following fixed order: the Mini-Mental State Examination (MMSE),^19^ Figure Memory Test (FMT),^15,16^ Semantic Verbal Fluency Test (VFT; animal category),^10,15^ Clock Drawing Test (CDT),^20^ Lawton IADL^21^ (answered by both elderly participant and a family member), Geriatric Depression Scale (GDS-15),^21,23^ and Pfeffer's Functional Activities Questionnaire (FAQ).^24^ The instruments are briefly described below.

*MMSE –* Screening test designed to quickly measure global cognitive functioning, temporal and spatial orientation, attention, immediate and short-term memory, language, praxis, and calculation. The cut-off points were established in accordance with the Brazilian study conducted by Brucki et al.^19^: 23 for 1-4 years of formal education; 26 for 5-9 years; and 27 for 10 years or more.

*Figure Memory Test (FMT) –* Evaluates episodic memory and consists of different tasks: naming and perception, incidental memory (IM), immediate memory (M1), learning (M2), delayed recall (M5), and recognition.^15,16^

*Verbal Fluency Test (VFT) - Animal Category –* Consists of saying as many animal names as quickly as possible in 1 minute. It assesses language, sustained attention, organization, strategy, and perseveration. The cut-off points were: 14 for <4 years of formal education, 15 for 5-9 years, and 16 for 10 years or more.^11,15,25^

*Clock Drawing Test (CDT) –* Cognitive screening tool that consists of drawing the face of a clock on a blank sheet of paper. It assesses praxis and visuoconstructive ability from a verbal command. Total score ranges from 1 to 10 points.^20^

*Lawton Instrumental Activities of Daily Living (IADL) Scale –* Assesses independent living skills. Eight functional domains are measured and individuals are scored according to their highest level of functioning in that category. A summary score ranges from 0 (low function, dependent) to 8 (high function, independent).^21^

*Geriatric Depression Scale (GDS-15) –* Assesses the degree of depressive symptoms (mild to severe). There are fifteen questions (yes or no answers) to describe how the subject has felt in the past two weeks. The cut-off points were: 5=normal; 5–10=mild depression; and >11=severe depression.^22,23^

*Pfeffer Functional Activities Questionnaire (FAQ) –* Evaluates the performance of elderly adults in activities of daily living through 10 questions. Each question is scored from 0 to 3. Total scores are on a scale ranging from 0 to 30. If the individual attains five points or more, they are defined as having impairment in instrumental activities of daily living.^24,26^

**Data analysis.** Initially, a descriptive analysis of the demographic, cognitive, and functional data was performed. Subsequently, data were assessed for normality. Since none of the variables were normally distributed, nonparametric tests were employed. First, Spearman's correlation analysis was employed for these same variables. The study sample was then stratified by age. Two age groups were established: Group 1 (60-74 years old) and Group 2 (≥75 years old). The groups were compared using the Mann-Whitney test for all of the cognitive and functional variables. Because this population was highly heterogeneous with regard to formal education (range: 1-24 years), the sample was also stratified by years of education: Group 1 (1-4 years), Group 2 (5-9 years), and Group 3 (≥10 years). The groups were then compared using the Kruskal-Wallis test. SPSS 16 software was used for all statistical analyses.

## RESULTS

Table 1 shows the sociodemographic characteristics of the sample. The sample consisted of elderly adults aged 60-96 years (age=72.75±6.92 years [mean±SD]), with 1-24 years of education (mean=9.28±4.79 years). The majority of the subjects were women and widows. A high level of education, an atypical characteristic for the Brazilian population, was also observed in the sample.

**Table 1:** Sociodemographic characteristics of the sample (n=264).

		Frequency (%)
Age	60-74 years	153 (58.0%)
	≥75 years	111 (42.0%)
Education	1-4 years	56 (21.2%)
	5-9 years	80 (30.3%)
	≥10 years	128 (48.5%)
Gender	Male	32 (12.1%)
	Female	232 (87.9%)
Marital status	Single	50 (18.8%)
	Married	54 (20.5%)
	Divorced	40 (15.2%)
	Widowed	120 (45.5%)

Table 2 shows the results of the participants' performance (mean, standard deviation, median and range) on the cognitive tasks that comprised the BCSB, GDS-15, and scales functional activities (Lawton IADL and Pfeffer FAQ).

**Table 2:** Performance on the BCSB cognitive tests (n=264).

Test	Mean (SD)	Median	Range
MMSE	24.73 (3.04)	25	15-30
VFT	15.89 (4.99)	16	3-30
CDT	6.33 (2.41)	5	0-10
Naming	9.87 (0.43)	10	7-10
Incidental memory	5.47 (1.34)	5.5	1- 9
Immediate memory	7.81 (1.47)	8	3-10
Learning	8.56 (1.33)	9	2-10
Delayed memory	7.58 (1.95)	8	1-10
Recognition	9.82 (0.56)	10	6-10
GDS-15	2.36 (2.63)	2	0-13
Lawton IADL	20.28 (1.25)	21	15-21
FAQ	0.89 (2.23)	0	0-12

BCSB: Brief Cognitive Screening Battery; SD: standard deviation; MMSE: Mini-Mental State Examination; CDT: Clock Drawing Test; VFT: Semantic Verbal Fluency Test (animal category). Tasks of Figure Memory Test: naming, incidental memory, immediate memory, delayed memory, learning and recognition. GDS-15: Geriatric Depression Scale; Lawton IADL: Lawton Scale; FAQ: Functional Activity Questionnaire. Number of subjects/family members who did not complete the tasks: CDT=2, GDS-15=2, Lawton=2, FAQ=110.

Descriptive analyses of the scales were performed to assess functioning and depressive symptoms in the sample. The mean (±SD) GDS-15 score was 2.36±2.64 (n=264), indicating that 17% of the sample had some symptoms of depression. Of these, 16.8% had symptoms of mild depression, and 1.5% had symptoms of severe depression. On the Lawton IADL scale, the mean score was 20.29±1.26(n=264), showing that participants were independent for activities of daily living. On Pfeffer's FAQ (i.e., another measure that assesses functioning), the mean score was 0.89±2.24 (n=156), showing that 6.4% of the sample had some kind of dependence for activities of daily living. Table 3 shows the results of the correlation analysis performed for scores on the cognitive tests, age, and education.

**Table 3:** Correlation (r) between BCSB measures, age, and education (n=264).

	Age	Education	GDS	Lawton	FAQ
MMSE	-0.90	0.41**	-0.1	0.20**	-0.28**
VFT	-0.10	0.41**	0.01	0.23**	-0.30**
CDT	-0.05	0.18**	-0.01	0.10*	0.02
Naming	0.09	0.11	-0.12	0.02	0.02
Incidental memory	-0.04	0.08	0.01	0.16*	-0.18*
Immediate memory	-0.01	0.06	-0.08	0.28**	-0.21**
Learning	-0.03	0.15*	-0.05	0.20**	-0.15*
Delayed memory	-0.10	0.16*	-0.04	0.30**	-0.17*
Recognition	-0.09	0.09	-0.08	0.19**	-0.01

BCSB: Brief Cognitive Screening Battery; MMSE: Mini-Mental State Examination; VFT: Semantic Verbal Fluency Test (animal category); CDT: Clock Drawing Test. Tasks of Figure Memory Test: naming, incidental memory, immediate memory, delayed memory, learning, and recognition. Two subjects did not complete the CDT.

* p<0.05,

** p<0.01, significant correlation.

Table 4 shows the results of the Mann-Whitney tests conducted between age groups for each BCSB task, the GSD-15, and the functional scales. No significant results were observed.

**Table 4:** Performance (mean [SD]) of the different age groups on the BCSB, GDS-15, and functional scales (n=264).

Test	60-74 years	≥75 years
MMSE	24.8 (3.2)	24.2 (3.2)
VFT	15.7 (5.2)	14.7 (4.4)
CDT	6.5 (2.4)	6.2 (2.3)
Incidental memory	5.5 (1.3)	5.3 (1.4)
Immediate memory	7.7 (1.5)	7.6 (1.5)
Learning	8.4 (1.4)	8.4 (1.3)
Delayed memory	7.5 (2.1)	7.3 (1.9)
Recognition	9.8 (0.7)	9.8 (0.6)
GDS-15	2.4 (2.5)	1.6 (2.5)
Lawton IADL	20.3 (1.3)	20.1 (1.5)
FAQ	0.1(2.5)	0.8(1.9

BCSB: Brief Cognitive Screening Battery; SD: standard deviation; MMSE: Mini-Mental State Examination; CDT: Clock Drawing Test; VFT: Semantic Verbal Fluency Test (animal category); GDS-15: Geriatric Depression Scale; Lawton IADL: Instrumental Activities of Daily Living; FAQ: Functional Activities Questionnaire. Tasks of Figure Memory Test: incidental memory, immediate memory, delayed memory, learningand recognition. Number of subjects/family members who did not complete the tasks: CDT=2, GDS-15=2, Lawton=2, FAQ=110. *p≤0.05, significant difference.

Table 5 shows the results of the Kruskal-Wallis test used to compare the education groups across the different instruments. For the functional scales, no differences were observed between groups on the Lawton IADL (p=0.523) or the GDS-15 (p=0.623). On Pfeffer's FAQ, however, significant differences were found between Groups 1 and 2 (p=0.006) and Groups 1 and 3 (p<0.001). Regarding the cognitive variables, differences were observed for learning, delayed memory, the CDT, VFT, and MMSE.

**Table 5:** Performance (mean [SD]) of the different education groups on the BCSB, GDS-15, and functional scales.

Test	1-4 years (G1)	5-9 years (G2)	≥10 years (G3)	Differences between groups
MMSE	22.3 (2.0)	24.3 (3.0)	26.0 (2.8)	G1<G2<G3**
VFT	12.6 (4.0)	14.4 (4.4)	17.3 (4.8)	G1<G2** G1<G3**
CDT	5.5 (2.7)	6.5 (2.4)	6.7 (2.1)	G1<G3*
Naming	9.8 (0.6)	9.9 (0.3)	9.9 (1.7)	
Incidental memory	5.2 (1.4)	5.6 (1.1)	5.5 (1.5)	
Immediate memory	7.6 (1.5)	7.6 (1.5)	7.8 (1.5)	
Learning	8.2 (1.6)	8.2 (1.3)	8.7 (1.3)	G2<G3*
Delayed memory	6.8 (2.3)	7.3 (1.8)	7.8 (1.8)	G1<G3* G2<G3*
Recognition	9.6 (1.1)	9.9 (0.3)	9.8 (0.4)	
GDS-15	2.6 (3.2)	2.0 (2.2)	2.0 (2.2)	
Lawton IADL	20.2 (1.4)	20.6 (0.8)	20.3 (1,2)	
FAQ	1.7 (2.8)	0.8 (2,4)	0.5 (1,6)	G1<G2** G1<G3**

BCSB: Brief Cognitive Screening Battery; MMSE: Mini-Mental State Examination; VFT: Semantic Verbal Fluency Test (animal category); CDT: Clock Drawing Test; Tasks of Figure Memory Test: incidental memory, immediate memory, delayed memory, learning and recognition. GDS-15: Geriatric Depression Scale; Lawton IADL: Instrumental Activities of Daily Living; FAQ: Functional Activities Questionnaire. n=264.

* p<0.05,

** p<0.01, significant difference.

## DISCUSSION

Toward understanding the transition from normal to pathological aging and providing an early diagnosis of neurodegenerative diseases, advances have been made in validating screening instruments to readily detect cognitive decline. However, studies describing cognitive and functional profiles of healthy community-dwelling older adults are scarce.^6,10,12,27^ Most studies are epidemiological and describe the prevalence and incidence of dementia, without presenting normative cognitive data of the healthy population.^26^ In this context, the aim of the present study was to describe a specific sample of independent community-dwelling elderly adults in Rio de Janeiro who participated in physical and cognitive activities offered by the government. The goal of the study, which is not epidemiological, was not to distinguish normal from pathological aging.

The BCSB is a precise battery used to diagnose cognitive impairment in heterogeneous populations. It has been employed in different contexts, including epidemiological studies,^4,10,12^ patients diagnosed with Alzheimer's disease,^4,8,14^ neurology outpatients with cognitive complaints and depressive symptoms,^28^ in geriatric clinics,^17,29^ and in individuals diagnosed with dementia in communities with a high prevalence of illiteracy.^16^ In the present study, the BCSB was used in a specific sample of community-dwelling elderly adults. The results indicated a pattern of normality with respect to cognitive and functional profiles because the performance was above the cut-off points when compared to normative studies in the literature.^10,11,14,19,25,30^

With regard to the sociodemographic characteristics of the sample, a high proportion of women and widowers was found, which is commonly observed in epidemiological studies in Brazil.^4,9,14,31^ The results confirmed a trend found in the literature, of a predominance of women, emphasizing the "feminization of old age".^1^ This demographic composition appears to be justified by the longer life expectancy of women in old age, which could be related to:

(i) lower exposure to occupational hazards;
(ii) lower mortality rates from external causes;
(iii) greater adherence to health services; and
(iv) greater participation in community activities and social groups at the location of sample recruitment.^1,9^ Great variability was found in formal education, with 47.5% of the sample having 10 years or more of education. This level of education differs from epidemiological population studies on aging conducted in smaller cities with data collection performed at participants' residences.^4,27^

This demographic profile appeared to have an impact on global cognitive performance, as observed on the MMSE. The mean MMSE score was similar to values reported by studies that evaluated cognitive functioning in healthy elderly adults^27^ and those comparing healthy older adults to individuals with MCI or dementia.^6,8,10,11,14,28,30,31,32^ In the present study, education significantly influenced MMSE scores. A significant increase in performance on the MMSE was found when comparing groups with low, middle, and high levels of education, similar to findings reported in the literature.^27^

Performance on the VFT was consistent with the cut-off point proposed by Charchat-Fichman et al.,^11^ confirming the preservation of semantic access ability, a rich vocabulary, processing speed, language, and executive function in this population. Educational level was associated with an increase in the number of words generated, especially among groups with low and high educational levels. These findings are consistent with previous studies.^5,10,12,27,29,30^ The mean VFT score in the present study was similar to that found in other studies evaluating healthy elderly adults. Performance on this test can be considered indicative of normal aging, for which it is highly sensitive in discriminating early stages of dementia.^5,6,10,14,28,32,33^

In addition to the MMSE and VFT, the sample's performance on the memory tasks was within the normal limits proposed by other Brazilian studies.^6,8,10,12,17,28,32^ The present study indicated that FMT performance was moderately associated with years of education, a result observed only for learning and delayed memory tasks. The results indicated an improvement in learning strategies and access to new stimuli with increasing education.

In the present study, the level of functioning indicated a healthy population that was autonomous and independent, where both these factors are required in IADL.^6,28^ This pattern of results contrasts with other studies.^17,29^ With regard to age, no difference in functional capacity for instrumental activities was found between the age groups. However, an association was found between performance on activities of daily living and education. Older adults with higher levels of education performed better. A study of active community-dwelling elderly adults found that more education and a higher income were related to greater engagement in intellectual, cultural, and leisure activities, which could justify the results in the present study.^5^ Other findings consistent with the literature were a strong association between instrumental activities of daily living and performance on cognitive tasks^11^.

However, on the CDT, the sample performed below expectations, but these results mirror those of some studies.^12,25^ The mean CDT score in the present study was 6.33±2.41, below the cut-off point established by other studies evaluating normal elderly adults.^6,8,10,28,32^ These results may indicate that the scoring criteria used for this task are too stringent, where participants predominantly scored 5. ^20^

Mendes-Santos^34^ used a subsample of 100 older adults derived from the present study and verified the applicability of the new CDT scoring criteria. A high percentage of the sample (53%) had a score of 5, thus corroborating the present findings. This performance can be interpreted as indicative of impairment in visuo-constructive and executive skills. There is clearly a need to develop new, more sensitive assessment standards that favor the planning and execution of the drawing in heterogeneous populations with regard to both age and education. Age negatively influenced performance on the task, whereas education positively influenced the results. These findings are consistent with the literature, which highlights the relationship between age and education in performance on the CDT and illustrates the variability of this task in a cognitively healthy elderly population^25,35^

The present results illustrate the cognitive profile of the sample. However, the behavioral data are also relevant to further understand this profile. Previous studies have reported a high incidence of depression among the elderly, which is influenced by biological and psychosocial variables.^8,27^ In this study, symptoms of depression were detected in 17% of the sample, and most of the symptoms were indicative of mild depression, according to the GDS-15 cut-off points, similar to other Brazilian studies involving community-dwelling older adults.^12,28^ Age did not correlate with depressive symptoms. However, there was a significant association with education. The severity of depressive symptoms measured by the GDS-15 was not associated with performance on any cognitive tasks in this particular sample.

Future studies should include illiterate older elderly individuals, with the aim of increasing our understanding of the heterogeneity that is inherent to the community-dwelling elderly population. Further studies should also more thoroughly investigate elderly individuals diagnosed with MCI and identify the prevalence of depression in cognitively and functionally healthy older adults.

A couple of limitations in the present study need to be mentioned. The first limitation refers to the exclusion of illiterate elderly adults from the sample. Another limitation is the possibility that participants at mild or very mild stages of dementia were also recruited, since the elderly adults enrolled in the activities of the community centers did not undergo a thorough geriatric or neurological evaluation, and diseases and medications were self-reported. These factors should be taken into account in future studies to further our understanding of the heterogeneity of the community-dwelling elderly population, evaluate individuals with MCI who do not attend geriatric services, and to identify the prevalence of depression in cognitively and functionally healthy elderly adults.

In summary, the present study sought to describe the performance of a community-dwelling sample of elderly adults in Rio de Janeiro on basic cognitive and instrumental activities of daily living, and depressive symptoms profile according to the GDS-15. Despite slightly lower-than-expected performance on the CDT, the results revealed a cognitive functioning profile compatible with a profile of normality and functional independence with regard to activities of daily living. These results contribute to a better understanding of the clinical concept of healthy aging and point to the relevance of participating in different physical, social, and cognitive activities for preventing diseases and improving health during the aging process.
